# Does Severe Maternal Morbidity Affect Female Sexual Activity and Function? Evidence from a Brazilian Cohort Study

**DOI:** 10.1371/journal.pone.0143581

**Published:** 2015-12-02

**Authors:** Carla B. Andreucci, José G. Cecatti, Rodolfo C. Pacagnella, Carla Silveira, Mary A. Parpinelli, Elton C. Ferreira, Carina R. Angelini, Juliana P. Santos, Dulce M. Zanardi, Jamile C. Bussadori, Gustavo N. Cecchino, Renato T. Souza, Maria H. Sousa, Maria L. Costa

**Affiliations:** 1 Department of Obstetrics and Gynaecology, School of Medical Sciences, University of Campinas, Campinas, SP, Brazil; 2 Federal University of Sao Carlos (UFSCar), Sao Carlos, SP, Brazil; Indiana University, UNITED STATES

## Abstract

**Objective:**

to assess Female Sexual Function Index (FSFI) scores and delay to resume sexual activity associated with a previous severe maternal morbidity.

**Method:**

This was a multidimensional retrospective cohort study. Women who gave birth at a Brazilian tertiary maternity between 2008 and 2012 were included, with data extraction from the hospital information system. Those with potentially life-threatening conditions and maternal near miss episodes (severe maternal morbidity) were considered the exposed group. The control group was a random sample of women who had had uncomplicated pregnancy. Female sexual function was evaluated through FSFI questionnaire, and general and reproductive aspects were addressed through specific questions. Statistical analyses were performed using Mann-Whitney and Pearson´s Chi-square for bivariate analyses. Logistic regression was used to identify variables independently associated with lower FSFI scores.

**Results:**

638 women were included (315 at exposed and 323 at not exposed groups). The majority of women were under 30 years-old in the control group and between 30 and 46 years-old in the exposed group (p = 0.003). Women who experienced severe maternal morbidity (SMM) had statistically significant differences regarding cesarean section (82.4% versus 47.1% among deliveries without complications, p<0.001), and some previous pathological conditions. FSFI mean scores were similar among groups ranging from 24.39 to 24.42. It took longer for exposed women to resume sexual activity after index pregnancy (mean 84 days after SMM and 65 days for control group, p = 0.01). Multiple analyses showed no significant association of FSFI below cut-off value with any predictor.

**Conclusion:**

FSFI scores were not different in both groups. However, they were lower than expected. SMM delayed resumption of sexual activity after delivery, beyond postpartum period. However, the proportion of women in both groups having sex at 3 months after delivery was similar. Altered sexual response may be evaluated as one of possible long-term consequences after SMM episodes. Further studies on the growing population of women surviving severe maternal conditions might be worth for improvement of care for women.

## Introduction

In 2013, the worldwide maternal mortality ratio (MMR) was 209 per 100,000 live births [1]. This is only a small part of the whole problem regarding the women´s health care during pregnancy, childbirth and postpartum period. For each woman dying, up to thirty others may experience severe complications that threaten their lives and/or cause any kind of temporary or permanent adverse consequences [2]. Women who almost died but survived after a severe obstetric complication are classified as “maternal near miss” according to the WHO (World Health Organization). This diagnosis is established through clinical, laboratory and/or management criteria [3].

Mapping severe maternal morbidity among cases of obstetric complications provides further information on prevalence and outcome of these conditions. Currently, SMM (severe maternal morbidity) and more specifically MNM (maternal near miss) are considered to be better indicators for maternal health condition than maternal mortality ratio alone [3, 4]. Nevertheless, there are not many studies addressing possible negative impacts of such conditions on women’s future wellbeing and health [5, 6].

In fact, little is known about long-term consequences on women’s physical or emotional health after an obstetric complication. Most studies on the subject followed these women no longer than forty-two days after childbirth. Appropriate understanding of the worsening of general health state following obstetric complications could improve quality of health care for this population.

Sexual function could theoretically be used to measure the impact of episodes of severe maternal morbidity on women´s life. However, there is limited data about the occurrence of sexual dysfunction comparing women with or without maternal morbidity. The Female Sexual Function Index (FSFI) is a validated instrument that addresses the subjectivity of sexual response by splitting sexual components (phases) into six domains [7]. Nevertheless, women who experienced severe maternal morbidity have not yet been properly studied regarding their sexual function. Between 60% and 90% of postpartum women are expected to resume sexual activity up to three months after an uncomplicated pregnancy [8]. Even so, there are not many studies addressing time to resumption of sexual intercourse after obstetric complications. Overall, it is not yet clear if severe maternal morbidity and near miss change female sexual response.

Therefore, the objective of this study was to assess Female Sexual Function Index (FSFI) scores and delay to resume sexual activity in association with severe maternal morbidity, in a cohort of women in Brazil.

## Method

### Setting and population

We conducted a multidimensional retrospective cohort study among women who gave birth between 1^st^ of July 2008 and 30^th^ of June 2012 at the maternity of the University of Campinas, in Brazil. This health facility is a tertiary referral unit in southeast Brazil. Female sexual function was one of several outcomes evaluated. Further aspects of health related conditions studied were general and reproductive health, disabilities, quality of life, posttraumatic stress disorder, substance abuse, and growth and development of children from the index pregnancy [5, 6]. These data are still to be published. These possible repercussions were assessed through validated questionnaires applied at two groups of women according to the exposure. The exposed group included women who presented potential life threatening conditions and/or maternal near miss episodes (both operationally defined as severe maternal morbidity) during pregnancy, childbirth and postpartum period, using the recent WHO definition and criteria [3]. The control (not exposed) group was a random sample of women who had had uncomplicated pregnancy, whose delivery occurred around the same time of each case. Time spent from delivery until interviews ranged from 6 months up to 5 years.

### Sample size estimation

The sample size was originally estimated for the whole cohort, considering all the outcomes to be evaluated and mainly the results for the disabilities and functioning using the WHO Disability Assessment Schedule (WHODAS 2.0) questionnaire. There are very few studies addressing the influence of severe maternal morbidity and/or maternal near miss on sexual aspects of women´s lives [9,10]. Specifically for aspects of sexual life, results of a previous cohort were used for sample size estimation [10]. Using the information of 43.1% of women who experienced maternal near miss referring any problems with sexual relations in comparison with 18.7% among women with uncomplicated pregnancies, a ratio of exposed to not exposed of 1:1, a type I error of 0.05 and type II error of 0.10, a total of 162 women would be necessary in each group. This is below the number currently evaluated.

### Selection of subjects

**Fig 1:**
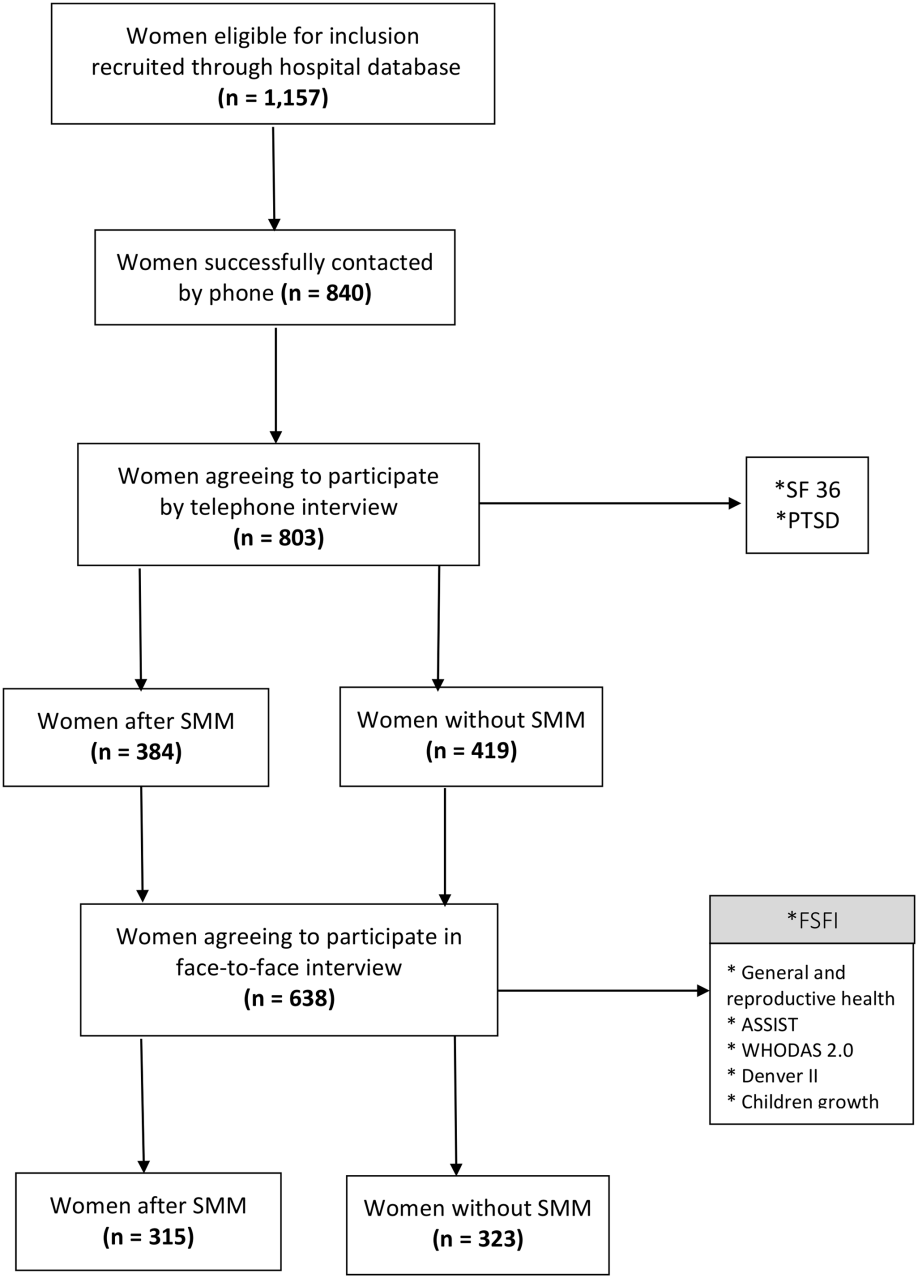
Flow chart for study inclusion.

Fig 1 describes procedures for the search of eligible participants. Through hospital electronic database, 1,157 women matched the selection criteria, i.e. either had had an episode of SMM or had delivered at the maternity without complications. All women who could be traced by telephone or mail letters were included and only non-responders were excluded. Using a system already available for telephone interview, 840 women were traced and contacted, what represented 72.6% of eligible subjects, and were invited to join the study by telephone. The 803 women who agreed to participate (95.6% of acceptance rate) recorded their agreement to the consent form read by the interviewer and then, were interviewed at that moment using the Computer Assisted Telephone Interview (CATI) system [11]. Thus, 384 exposed and 419 not exposed women answered the questionnaires SF 36 (Quality of Life) and PTSD (Posttraumatic Stress Disorder). In addition, by the end of telephone conversation, the participating women were invited to come for a face-to-face visit at the hospital when they should also bring their children (from the specific pregnancy being assessed) for evaluation. The second interview was scheduled at women´s convenience with expenses covered. Out of 803 participants that were initially recruited, 638 came to the second stage, which conferred the continuity rate of 79.5%. Among those women, 323 were controls, and 315 had experienced severe maternal morbidity, including 67 maternal near miss cases. The 638 women who answered the FSFI compared with those 165 initially eligible but not coming for the interview showed no significant different results for maternal age, parity, mode of delivery, sex of the child and neonatal outcome (data not shown).

### Data collection

During hospital interviews, after new written informed consents for the women and their children were read and signed, we obtained information about reproductive and general health of women before and after the index gestation, and applied validated questionnaires for addressing all the objectives of the whole study. For general health and reproductive aspects assessment, a specific instrument was developed. Along with obstetric background, questions covered also time to resume sexual activity after index pregnancy. We applied the Female Sexual Function Index (FSFI) for assessing female sexual response, comparing each six-domain scores and the total FSFI scores (mean, SD and median) between both groups. At this point, WHODAS 2.0 questionnaire [12], Denver Developmental Screening Test [13], and the standard WHO questionnaire ASSIST for drug use [14] were also applied in these women or their children, plus the measurement of weight and height of children. These results will be further detailed explored.

### Data management and analyses

Finally, collected data were manually transcript on previously printed forms, and later digitally included in an electronic database built with LimeSurvey^®^. Further, data were transferred to SPSS files and, before analysis, detailed multiple processes of checking data consistency were developed. For each inconsistency found, the original form and/or the correspondent clinical records were re-checked to correct the database. If this was not enough, the woman was contacted again by telephone to check the information. This process was repeated as many times as necessary to obtain a clean database with no inconsistencies. Non-parametric Mann-Whitney and Pearson´s or Yates Chi-square tests were used to compare general characteristics of women, time to resume sexual activity and FSFI mean and median scores in both groups. The cut-off value for FSFI to be considered low was 26.55 (7). Poisson multiple regression analysis was performed to identify variables independently associated with lower FSFI scores. The report was prepared following the recommendations of the STROBE statement [15]. The database is available in S1 File.

### Ethical aspects

The study was approved by the Institutional Review Board of the University of Campinas and was sponsored by the CNPq (Brazilian National Research Council), which played no other role on the development of the study, data collection, analyses and the report. Women had two inform consent forms, one for the first step of the study performed by telephone where they orally gave their consent that was recorded, and the second for the face-to-face interview with them and their children, which they read and signed.

## Results

Table 1 shows sociodemographic, pregnancy and perinatal characteristics of included women. Age was significantly different in both groups. The majority of women in the control group were under 30 years-old (52%), and 61% in exposed women were between 30 and 46 years-old. Cesarean section was more frequently performed among women with severe maternal morbidity (82.5%), compared with women in the control group (47.1%). Women who developed severe maternal morbidity had also more prevalent previous pathological conditions, such as hypertensive disorders (23% versus 6.5%), obesity (21% versus 11%), diabetes (7% versus 3%), cardiac diseases (5.5% versus 1%), respiratory diseases (5% versus 1%), thyroid dysfunction (6.7% versus 2.5%), neurologic (4.1% versus 1.2%) and renal diseases (3.5% versus 0.3%). Smoking was also more common among exposed women (5.7% versus 0.9%). There were no differences between groups regarding number of pregnancies, ethnicity, years of schooling, or perinatal outcomes. Having a partner and time of breastfeeding were also similar in both groups.

**Table 1:** Socio demographic, pregnancy and perinatal characteristics of women according to maternal morbidity. SMM: severe maternal morbidity. Values in bold mean that they are statistically significant (p<0.05).

Characteristics	Group		
	SMM	No SMM	p-value*
**Maternal age (y)**	**n (%)**	**n (%)**	**0.003**
≤ 19	10 (3.2)	19 (5.9)	
20–29	113 (35.9)	149 (46.1)	
30–39	146 (46.3)	128 (39.6)	
≥ 40	46 (14.6)	27 (8.4)	
**Number of pregnancies**			0.778
1	102 (32.4)	109 (33.7)	
≥2	213 (67.6)	214 (66.3)	
**Ethnicity**			0.086
White	152 (48.3)	133 (41.2)	
Non white	163 (51.7)	190 (58.8)	
**Schooling (years)** ^**a**^			0.295
Up to 8	102 (32.5)	91 (28.3)	
Above 8	212 (67.5)	230 (71.7)	
**Marital status** ^**b**^			0.871
With partner	259 (82.5)	269 (83.3)	
No partner	55 (17.5)	54 (16.7)	
**Time since delivery (y)**			0.096
< 1	45 (14.3)	40 (12.4)	
1–<2	95 (30.2)	127 (39.3)	
2–<3	107 (34.0)	101 (31.3)	
≥ 3	68 (21.6)	55 (17.0)	
**Route of delivery** ^**a**^			**<0.001**
Vaginal	55 (17.6)	171 (52.9)	
Cesarean section	257 (82.4)	152 (47.1)	
**Perinatal outcome** ^**c**^			0.087
Alive	261 (95.3)	309 (98.1)	
Neonatal death	13 (4.7)	6 (1.9)	
**Previous conditions**			
Chronic hypertension	72 (22.9)	21 (6.5)	**<0.001**
Morbid obesity	65 (20.6)	36 (11.1)	**0.002**
Diabetes	21 (6.7)	9 (2.8)	**0.033**
Smoking	18 (5.7)	3 (0.9)	**0.002**
Cardiac diseases	17 (5.4)	3 (0.9)	**0.003**
Respiratory diseases	16 (5.1)	4 (1.2)	**0.011**
Thyroid diseases	21 (6.7)	8 (2.5)	**0.019**
Neurologic diseases	13 (4.1)	4 (1.2)	**0.043**
Renal diseases	11 (3.5)	1 (0.3)	**0.008**
**Total**	**315**	**323**	

*Pearson Chi-square test for tables greater than “2x2” and Yates Chi-square test for tables “2x2”.

Missing information for a: 3; b: 1; c: 49 cases.

The mean and median FSFI scores were similar in the population studied. After either an episode of severe maternal morbidity (MNM or PLTC) or pregnancies without complication, total FSFI scores were respectively 24.39 and 24.42. Specific domains of FSFI questionnaire also showed no differences among groups (Table 2).

**Table 2:** Mean and median values for FSFI total and domain scores according to maternal morbidity. SMM: severe maternal morbidity; PLTC: potentially life threatening condition; MNM: maternal near miss. Values in bold mean that they are statistically significant (p<0.05).

Morbidity	Mean	SD	Median	Min	Max	n	p-value *
**Desire domain**							0.684
SMM	3.33	1.28	3.60	1.2	6.0	315	
No morbidity	3.37	1.17	3.60	1.2	6.0	323	
**Arousal domain** ^**a**^							0.438
SMM	3.40	1.77	3.90	0.0	6.0	312	
No morbidity	3.55	1.65	3.90	0.0	6.0	323	
**Lubrication domain** ^**b**^							0.604
SMM	4.03	1.97	4.50	0.0	6.0	310	
No morbidity	4.14	1.88	4.50	0.0	6.0	318	
**Orgasm domain** ^**a**^							0.999
SMM	3.78	2.01	4.40	0.0	6.0	312	
No morbidity	3.87	1.86	4.40	0.0	6.0	323	
**Satisfaction domain** ^**c**^							0.635
SMM	4.43	1.53	4.80	0.8	6.0	287	
No morbidity	4.42	1.47	4.80	0.8	6.0	305	
**Pain domain** ^**a**^							0.115
SMM	4.00	2.10	4.60	0.0	6.0	312	
No morbidity	4.32	1.93	4.80	0.0	6.0	323	
**Total FSFI Score** ^**d**^							0.937
SMM	24.39	8.02	26.80	1.8	35.7	282	
PLTC	24.79	7.87	26.90	1.8	35.7	223	
MNM	22.87	8.47	24.10	2.0	34.5	59	
No morbidity	24.42	7.80	26.50	2.0	36.0	301	

* Nonparametric test: Mann-Whitney

Missing information for a: 3; b: 10; c: 46; d: 55 cases

Moreover, we found no association between FSFI scores and women´s individual or pregnancy characteristics (Table 3). After either severe maternal morbidity or uncomplicated childbirth, total FSFI scores did not significantly differ at same age or parity, years of schooling, marital status, ethnicity, route of delivery, or perinatal outcome. Not exposed women under 19 years-old had the highest total FSFI mean score (27.53), while those without a partner had the lowest total FSFI mean score (19.04), however also not significantly different from those with SMM. Finally, time elapsed between delivery and study interviews were not correlated to FSFI total mean scores at both exposed and control group.

**Table 3:** Mean values of FSFI scores according to maternal morbidity by some maternal and delivery characteristics. Values in bold mean that they are statistically significant (p<0.05).

Characteristics	Morbidity	Mean	SD	n	p-value *
**Maternal age (y) ^a^**					
≤ 19	SMM	23.67	9.23	9	0.375
	No morbidity	27.53	3.45	18	
20–29	SMM	25.27	7.68	102	0.280
	No morbidity	24.26	7.94	141	
30–39	SMM	23.94	7.99	131	0.575
	No morbidity	24.41	7.96	116	
≥ 40	SMM	23.79	8.78	40	0.637
	No morbidity	23.24	8.35	26	
**Number of pregnancies** ^**a**^					
1	SMM	25.05	7.75	91	0.862
	No morbidity	24.79	7.88	102	
≥ 2	SMM	24.08	8.15	191	0.998
	No morbidity	24.23	7.78	199	
**Schooling (years)** ^**b**^					
Up to 8	SMM	23.41	7.90	93	0.789
	No morbidity	23.69	7.69	83	
Above 8	SMM	24.87	8.06	189	0.648
	No morbidity	24.66	7.87	216	
**Ethnicity** ^**a**^					
White	SMM	24.31	7.99	133	0.427
	No morbidity	24.77	8.20	122	
Nonwhite	SMM	24.47	8.08	149	0.430
	No morbidity	24.19	7.54	179	
**Marital status** ^c^					
With a partner	SMM	24.88	7.54	247	0.922
	No morbidity	25.25	6.53	261	
Without a partner	SMM	20.70	10.34	34	0.970
	No morbidity	19.04	12.23	40	
**Time since delivery (y)** ^**a**^					
< 1	SMM	24.88	7.56	41	0.519
	No morbidity	24.72	7.61	37	
1–<2	SMM	24.66	7.80	89	0.533
	No morbidity	24.13	7.73	117	
2–<3	SMM	23.95	9.09	95	0.677
	No morbidity	24.80	8.20	95	
≥ 3	SMM	25.07	6.82	57	0.678
	No morbidity	24.19	7.56	52	
**Route of delivery** ^**b**^					
Vaginal	SMM	24.97	7.61	54	0.959
	No morbidity	24.78	7.90	157	
Cesarean section	SMM	24.28	8.15	226	0.535
	No morbidity	24.04	7.71	144	
**Child outcome** ^d^					
Alive	SMM	24.63	7.61	235	0.695
	No morbidity	24.33	7.76	288	
Neonatal death	SMM	20.02	10.75	11	0.583
	No morbidity	25.92	5.53	5	

* Nonparametric test: Mann-Whitney

Missing information for a: 52 cases; b: 57; c: 56; d: 99 cases.

Although the rates of FSFI scores <26.55 were not different within groups considering all women or only those reporting sexual activity during the past 4 weeks, exposed women took longer to resume sexual activity after index pregnancy (mean of 84 days for women with SMM and 65 days for those without complications, p = 0.011). This delay was significant at 45 days postpartum, but no longer at 90 days. Additionally, when the outcome of child survival was taken into account, the mean time to resume sexual activity was higher for women with SMM and an alive child, while this difference was not significant for those who had a neonatal death. The main reasons mentioned for postponing sexual activity were lack of partnership, lack of interest, fear of being hurt, tiredness, and fear of getting pregnant again. Although no significant differences were detected in both groups regarding these reasons, the fear of being hurt among women with SMM was almost the double of those given by women with uncomplicated pregnancies (Table 4).

**Table 4:** Proportion of women identified as having FSFI total score <26.55, time to resume sexual activity and reasons for delaying resumption of sexual activity according to maternal morbidity. Values in bold mean that they are statistically significant (p<0.05).

Characteristics	Group		
	SMM	No SMM	p-value
**Total FSFI score ^a^**	**n (%)**	**n (%)**	
**Only sexually active women**			
< 26.55	109 (41.9)	128(46.2)	0.361*
≥ 26.55	151 (58.1)	149 (53.8)	
**All women**			
< 26.55	131 (46.5)	152 (50.5)	0.372*
≥ 26.55	151 (53.5)	149 (49.5)	
**Time to resume sexual activity (days)** ^**b**^			
≤ 45	147 (49.2)	184 (59.5)	**0.013** *
> 45	152 (50.8)	125 (40.5)	
≤ 90	252 (85.1)	276 (89.8)	0.072*
> 90	47 (14.9)	33 (10.2)	
**Mean time (± SD)** ^**b**^	83.69 (±103.85)	65.39 (±81.62)	**0.011** **
**Mean time to resume sexual activity X Child outcome (days)** ^**c**^			
Child alive (n = 547)	86.3 (±110.2)	66.3 (±83.3)	**0.019** **
Neonatal death (n = 18)	73.3 (±61.1)	44.7 (±15.4)	0.437**
**Total**	**315**	**323**	
**Reasons for delaying resumption of sexual activity beyond 90 days**			#
No partner	20 (42.6)	16 (48.5)	
Lack of interest	13 (27.7)	8 (24.2)	
Fear of being hurt	11 (23.4)	5 (15.2)	
Tiredness	2 (4.3)	2 (6.1)	
Fear of getting pregnant	1 (2.1)	2 (6.1)	
**Total**	**47**	**33**	

*Yates Chi-square test.

**Mann-Whitney test.

# Test not applicable.

Missing information for a: 55; b: 30; c: 73 cases.

The mean time to resume sexual activity was not influenced at all by parity (a proxy of number of young children living together), time since delivery (when a recall bias could be possible), and by breastfeeding status. Only the absence of a partner showed to be associated with a longer time (Table 5), although without differences in scores between groups. Finally, logistic regression analyses showed no association between FSFI below cut-off values with any predictor assessed.

**Table 5:** Mean time to resume sexual activity according to some characteristics.

Characteristics	Mean	SD	p-value
Parity			0.554
1	76,04	79,87	
≥ 2	73,59	99,62	
**Marital status**			<0.001
Without a partner	137,82	165,71	
With a partner	63,60	69,65	
**Time since delivery (y)**			0.540
< 1	74,89	73,99	
1–<2	63,22	55,37	
2–<3	79,13	106,66	
3 ->3	86,28	129,63	
**Breastfeeding status**			0.775
Yes	73,60	94,77	
No	77,94	88,79	

## Discussion

Our findings show that women in the severe maternal morbidity group were older, with higher cesarean section rates, and had more previous pathological conditions than women in the control group. In addition, there were no differences at all in the total and each domain FSFI scores between women who had severe maternal morbidity and those without maternal complications. However, women experiencing SMM took longer than those without complications to resume sexual activity in the postpartum period, independently of the outcome of the child, parity, breastfeeding status or time since delivery. Multiple logistic regression analysis revealed no association of low scores of FSFI with any predictor. Some previous studies described correlation of sexual dysfunction and maternal age, years of schooling, ethnicity and mode of delivery. However, these findings presented conflicting conclusions [16–19]. To the best of our knowledge, no other previous study addressed sexual functioning using validated questionnaires among women with severe maternal morbidity.

Although sexual dysfunction is recognized to be a health disorder [20, 21], the screening and diagnosis of this condition is not easy and is not a routine practice, especially in association with childbirth. When compared with women who had had an uncomplicated birth, those who underwent episodes of severe maternal morbidity delayed resumption of sexual activity. Accordingly, time to resume sexual intercourse after index pregnancy was meanly 18 days longer at the exposed women. Furthermore, differences between groups were significant at 45 days postpartum, which provides evaluation beyond classic postpartum period definition (up to 42 days). Possible pregnancy or childbirth repercussions are usually examined only at that time span, and knowledge on long-term burden is very limited. Even without obtaining significant difference between groups at 90 days after childbirth, our findings show that SMM group delayed to resume sexual activity beyond postpartum period, as was the case also for women without a partner, what is quite understandable. This delay was not shown to be associated with neonatal outcome as found by a previous study, although the current numbers are perhaps too low for definitive conclusions on this regard [9]. In addition, having a partner, and therefore being sexually active, and time of breastfeeding were not associated with FSFI total scores.

Additionally, “fear of being hurt” was more frequently mentioned as a reason for delaying resumption of sexual activity among exposed women (23.4% versus 15.2%). Although statistical analysis was not feasible, the finding may possibly imply correlation between maternal morbidity and longer or more intense soreness after delivery. Even more, specific SMM involving ICU admission or surgery recovery could lead to slower healing processes and more intense pain perception, as well as emotional repercussions, which has been described related to termination of pregnancy [22].

On the other hand, we found no differences among groups regarding domains or total FSFI scores. Although the Female Sexual Function Index questionnaire is widely applied as a sexual function-screening tool, this evaluation is limited. The FSFI may not contain all possible emotional and psychological elements needed to identify altered female sexual response. Despite being validated to address subjectivity, the FSFI questionnaire assesses some biological aspects of sexual response. Even the “arousal” component might be influenced by hormone levels, which are notably lower during the postpartum period [23]. This particular component defined the instrument´s construct validity [7]. In addition, there is high prevalence of suspected sexual dysfunction in general female population, regardless age or any chronic disorders [16, 24–26]. Although several conditions were evaluated as exposure for sexual dysfunction in Brazil, specific cut-off values for the FSFI questionnaire had not yet been tested [24, 27–30]. As a result, the originally established cut-off value for total FSFI scores is also applied in Brazilian studies, as was the case also for the current one.

Nevertheless, the total FSFI scores among all women were lower than expected. In all groups, total mean scores ranged from 22.87 to 24.79, when the cut-off value considered for suspected sexual dysfunction was below 26.55 [31]. Mean and median FSFI scores also did not differ among included women. However, median total score of 24.10 in the maternal near miss sub-group was the only below the cut-off value 26.55. Despite not significant, this finding may contribute to map possible long-term repercussions on these women´s lives.

Moreover, sexual satisfaction is acknowledged as a quality of life measurement parameter. A previous study described 43% of prevalence of sexual problems among women after severe maternal morbidity and its correlation with depressive symptoms. This prevalence is however, the same described for the overall female population in USA. Overall, exposed women had poorer general health condition [10, 16]. The impact of uncomplicated pregnancy and delivery related to altered female sexual response has also been previously described [19, 32, 33]. Therefore, it is reasonable to assume that severe obstetric morbidity might also contribute to modify such aspects of women´s lives. Together with delay to resumption of sexual activity, suspected sexual dysfunction on women who experienced MNM might suggest correlation between these conditions and altered self-perception regarding quality of life. Additionally, despite being high, maternal death ratio has been dropping for several years. Consequently, there is a growing population of SMM and MNM surviving women who were so far not properly evaluated.

The current study has of course some possible limitations. Although this was a cohort study, evaluation of outcomes might be limited because of the retrospective way the data were collected. Ideally in a prospective follow-up, the women should be followed and information regarding sexual activity and function should be collected at predetermined regular periods after delivery. In this study, information on sexual aspects was collected just once in different times of postpartum period, however no differences at all were found in relation to the time since delivery, suggesting a lower likelihood of an important recall bias effect. This was a multidimensional study, and was not specifically addressed to evaluate completely the female sexual response. Therefore, some relevant variables of sexual life were not included, such as information on lactational amenorrhea, social support for breastfeeding or family contexts. Thus, a prospective evaluation is proposed for the next research step approaching this topic. Furthermore, there are not specific standardized cut-off values for the FSFI score to be applied among Brazilian women and, in addition, after severe maternal morbidity this has never been performed before.

Our study showed that women who experienced episodes of severe maternal morbidity delayed resumption of sexual activity, although FSFI did not differ between groups. FSFI scores had not been compared before among women with or without severe morbidity. The absence of statistically significant differences between exposed and not exposed groups might be correlated to high prevalence of altered FSFI scores, perhaps due to questionnaire´s limitations. Nevertheless, all FSFI mean total scores were below the cut-off value. Moreover, sexual dysfunction, as well as a broader evaluation of sexual life aspects should be further and in-depth studied among women who survived life-threatening conditions.

## Supporting Information

S1 File FSFI-Database. (XLS)
